# The Representativeness of Outdoor Particulate Matter Concentrations for Estimating Personal Dose and Health Risk Assessment of School Children in Lisbon

**DOI:** 10.3390/ijerph20085564

**Published:** 2023-04-18

**Authors:** Eleftheria Chalvatzaki, Sofia Eirini Chatoutsidou, Susana Marta Almeida, Lidia Morawska, Mihalis Lazaridis

**Affiliations:** 1 School of Chemical and Environmental Engineering, Technical University of Crete, Chania 73100, Greece; echalvatzaki@tuc.gr (E.C.); sochatoutsidou@tuc.gr (S.E.C.); 2 Centro de Ciências Tecnologias Nucleares, Instituto Superior Técnico, Universidade de Lisboa, Estrada Nacional 10, Km 139.7, 2695-066 Bobadela LRS, Portugal; 3 School of Earth and Atmospheric Sciences, Queensland University of Technology, 2 George Street, Brisbane, QLD 4000, Australia

**Keywords:** personal dose, particulate matter, fixed monitoring station, school children

## Abstract

This study investigated the suitability of outdoor particulate matter data obtained from a fixed monitoring station in estimating the personal deposited dose. Outdoor data were retrieved from a station located within the urban area of Lisbon and simulations were performed involving school children. Two scenarios were applied: one where only outdoor data were used assuming an outdoor exposure scenario, and a second one where an actual exposure scenario was adopted using the actual microenvironment during typical school days. Personal PM_10_ and PM_2.5_ dose (actual exposure scenario) was 23.4% and 20.2% higher than the ambient (outdoor exposure scenario) PM_10_ and PM_2.5_ doses, respectively. The incorporation of the hygroscopic growth in the calculations increased the ambient dose of PM_10_ and PM_2.5_ by 8.8% and 21.7%, respectively. Regression analysis between the ambient and personal dose showed no linearity with R^2^ at 0.07 for PM_10_ and 0.22 for PM_2.5_. On the other hand, linear regression between the ambient and school indoor dose showed no linearity (R^2^ = 0.01) for PM_10_ but moderate (R^2^ = 0.48) for PM_2.5_. These results demonstrate that ambient data must be used with caution for the representativeness of a realistic personal dose of PM_2.5_ while for PM_10_ the ambient data cannot be used as a surrogate of a realistic personal dose of school children.

## 1. Introduction

National environmental agencies monitor ambient particulate matter (PM) concentrations using fixed monitoring stations, which are located in various locations representing different types of environments (e.g., traffic, urban, background, etc). These data are often used to assess exposure in epidemiological studies [1]. 

There are several studies proposing that data from fixed monitoring stations are poor predictors for personal exposure [2,3,4], while other studies suggest that outdoor data can be treated as a surrogate for personal exposure in epidemiological studies [4,5,6]. Kousa et al. [3] asserted that ambient PM concentrations are poor predictors of personal exposure due to personal movement in different microenvironments, however, ambient PM concentrations correlate quite well with residential indoors in the absence of significant indoor sources. Hence, they are more representative of the personal exposure of individuals who stay at home with very limited activities (e.g., babies and the elderly). Kim et al. [6] asserted that PM_2.5_ data from a fixed monitoring station can be treated as a surrogate for personal exposure of an individual who lives at a close distance (median distance ≈ 4 km). Likewise, Zhang et al. [4] and Borgini et al. [5] pointed out that ambient PM_2.5_ measurements can be used as an accurate indicator for the personal exposure of school children. Contrary, Zhang et al. [4] observed that for PM_10_ the correlation was weak. Regarding ultrafine particles (UFP), Pradhan et al. [7] found that the daily average particle number (PN) concentrations from a monitoring station can be used to estimate the daily average personal exposure whereas the opposite finding was observed for hourly average PN concentrations. Additionally, Gu et al. [2] found that the PN concentration of UFPs at a monitoring station is a poor predictor of personal exposure for both short-term (1 min data) and long-term periods due to traffic and indoor sources. The representativeness of ambient concentrations for the determination of personal exposure varies between different particle metrics (PM and PN), particle size (PM_10_ and PM_2.5_), population groups, and scientific studies. Indoor particles provide a major contribution to the total exposure as they can affect human health with 10–30% of the total burden of disease [8]. The non-inclusion of indoor particle sources causes inaccurate personal exposure estimations and poor quantification in health estimations [9]. Therefore, the inclusion of indoor contributions to the estimation of personal exposure is important.

The total exposure of children is significantly affected by the school microenvironment due to their everyday exposure [10]. PM levels in classrooms can be affected by many factors such as outdoor penetration, quality of ventilation, chalk emissions, painting, movement/activities of children, cleaning activities, and occupancy [11,12,13,14,15,16]. The poor air quality inside classrooms is linked with severe respiratory diseases (e.g., asthma, allergic rhinitis) and low lung function, especially for those children in highly polluted schools [17,18]. Therefore, air quality evaluation in schools is important due to the high PM levels and the associated health effects, which constitute the necessary development of control plans and mitigation strategies.

Epidemiological studies show associations between PM concentrations and increased morbidity and mortality [19,20]. Specifically, Pope et al. [19] asserted that a 10 μg/m^3^ decrease in PM_2.5_ concentration is linked with an increase in life expectancy of 0.61 ± 0.20 years. Additionally, Pope and Dockery [20] found that an increase of 10 μg/m^3^ in PM_2.5_ concentration is associated with a 1% increase in mortality. Exposure to PM_2.5_ can lead to cardiovascular disease mortality, non-fatal events, and a decrease in life expectancy [21]. In addition, the relative risk for all non-accidental mortality was equal to 1.04 and 1.08 per 10 μg/m^3^ increase in annual PM_10_ and PM_2.5_ concentrations, respectively [22]. Therefore, an increase of 10 μg/m^3^ in annual PM_10_ and PM_2.5_ concentrations is associated with a 3.8% and 7.4% increase in mortality, respectively. Regarding short-term exposure, the relative risk for all non-accidental mortality was equal to 1.0041 and 1.0065 per 10 μg/m^3^ increase in PM_10_ and PM_2.5_ concentrations, respectively [22]. Therefore, a 10 μg/m^3^ increase in PM_10_ and PM_2.5_ concentrations is associated with a 0.4% and 0.6% increase in mortality, respectively. This study aims to investigate the representativeness of PM concentrations obtained from a fixed monitoring station in the estimation of the received dose for school children in Lisbon. Previous studies in the scientific literature focused on the use of ambient concentrations as an indicator for personal exposure whereas the innovation of the current study is the investigation of the suitability of ambient data from a fixed monitoring station for estimating the personal dose that takes into consideration the hygroscopicity of particles. For this purpose, two different approaches were applied: firstly, the dose was estimated only by outdoor data assuming a 24 h outdoor exposure scenario, and secondly, the dose was estimated using the actual exposure scenario taking into consideration the different microenvironments where children spent their time. In both cases, a dosimetry model (ExDoM2; [23,24]) was used to estimate the deposited dose for different regions of the respiratory tract along with an evaluation of the health risk assessment by exposure to both PM scenarios.

## 2. Materials and Methods

### 2.1. Study Area

Lisbon is the capital of Portugal with the population of a metropolitan area of 2.82 million inhabitants representing approximately 27% of Portugal’s population. Besides the input of air pollutants from urban sources, Lisbon has a significant contribution to the marine environment due to the city’s geographic position along the Atlantic coast [25]. The selected monitoring station is located in the area of Olivais in eastern Lisbon and is characterized as an urban background site (38.7698, −9.10729) [26]. Particularly, the station (Olivais) is situated far from the main roads but at 2.5 km, in the east direction from the airport and at 1.5 km in the west direction from the Tagus Estuary in the city of Lisbon. The station at Olivais was selected as a representative outdoor monitoring station due to its proximity to the under-study region.

**Figure 1:**
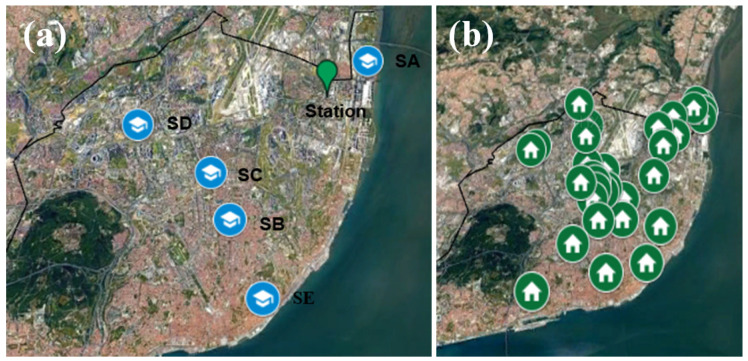
(**a**) Locations of the schools and fixed monitoring station and (**b**) location of houses.

The schools are spread in the area around the monitoring station (Figure 1a) with the closest one at a distance of 1.5 km northeast of the station and near the coast (school SA). On the other hand, the remaining 4 schools (SB, SC, SD, and SE) are located in the southwest direction from the station at varying distances (4.5–6.5 km) with school SD located nearby a highway characterized by significant vehicular traffic, whilst, school SE is also located close to the Tagus Estuary. Lastly, the locations of the test houses cover the urban area all around the station and the schools as shown in Figure 1b.

### 2.2. Data Origin

Ambient PM_2.5_ and PM_10_ concentrations were derived from Qualar, which is the online information system, developed by the Portuguese Environment Agency (APA), with the aim of centralizing all information regarding air quality measurements carried out by the Portuguese air quality monitoring network [27]. In this work, we considered the urban background station Olivais, which is equipped with reference instruments that measure PM_2.5_ and PM_10_ concentrations using beta attenuation technology (Environment MP101M). 

On the other hand, indoor PM_2.5_ and PM_10_ concentrations correspond to already published data conducted through field campaigns in 5 schools and 34 houses within the metropolitan area of Lisbon [13,25]. Accordingly, field PM_2.5_ and PM_10_ measurements at the sampling sites (schools and houses) took place between September 2017 and July 2018 and during the teaching/occupation time (8 h in schools and 15 h in houses) [12,13,25]. In particular, sampling at the houses was performed during variable periods along the duration of the campaign, whereas, explicit sampling periods correspond for each school as presented in Table 1. Mass concentrations (PM_2.5_ and PM_10_) were measured with a Leckel sampler (MVS6; Sven Leckel, Germany) at a constant flow rate of 2.3 m^3^/h whilst a Sioutas impactor was used (see Figure S1 in supplementary material) for the collection of size-segregated mass fractions (<0.25 μm, 0.25–0.5 μm, 0.5–1 μm, 1–2.5 μm). Detailed descriptions of sampling protocols can be found by Faria et al. [13] and Martins et al. [25]. PM_2.5_ size distribution was taken from Sioutas measurements whereas coarse particle concentration in the size class 2.5–10 μm was obtained from Leckel measurements. In each stage, the particle size distribution was considered monodisperse (σ_g_ = 1) and hence the geometric midpoint (square root of lower cut-off size × upper cut-off size) was used for the calculations.

### 2.3. Exposure Methodology

Dosimetry calculations were performed for 10-year-old children that are exposed to PM concentrations during typical school days (Monday–Friday). The subject was considered a nose breather under varying physical exertion levels (sleep, sitting, and light exercise). Two scenarios were applied in the current study (Table 1). Accordingly, in the first approach, dosimetry calculations were performed assuming an outdoor exposure scenario, whereby the students were considered to spend their day entirely outdoors (24 h exposure) and outdoor data from the fixed monitoring station were incorporated into the model and for the sampling period that corresponds to each school. Alternatively, in the second approach, dosimetry calculations were performed using the actual exposure scenario involving three different microenvironments (house indoors, school indoors, and school outdoors) according to a daily activity profile (Table S1 in supplementary material) that was obtained from questionnaires [13]. Overall, dosimetry calculations were performed individually for each one of the five schools considering the same house microenvironment in the respective house hours, with an average concentration estimated by all 34 houses.

### 2.4. Dosimetry Model

ExDoM2 [23] uses the semi-empirical equations of the International Commission on Radiological Protection (ICRP) [28,29] for the simulation of deposition of particles in the human respiratory tract and was recently extended in order to incorporate the hygroscopic growth of particles by adopting the κ-Köhler theory [24].

The deposition fraction was calculated for nine filters, which correspond to two filters for the anterior nose (ET1) region; two filters for the posterior nasal passages, pharynx, and larynx (ET2) region; two filters for the bronchial (BB) region; two filters for bronchiolar (bb) region and one filter for the alveolar-interstitial (AI) region. The tracheobronchial region (TB) is the sum of BB and bb regions. The deposition fractions were calculated with the following equation [28]:

(1) $${DE}_{j}=n_{j}\phi_{j}\prod_{jj=0}^{j-1}\left(1-n_{jj}\right)$$

where $n_{j}$ is the deposition efficiency of the j filter, $\phi_{j}$ is the fraction of tidal air that reaches the j filter and $n_{0}$ is the prefiltration efficiency.

The prefiltration efficiency (n_0_) was estimated with the following equation [28]:

(2) $$n_{0}=1-n_{I}$$

where n_I_ is the inhalability of particles.

The deposition efficiency (n_j_) was estimated by [28]:

(3) $$n_{j}={\left(n_{ae}^{2}+n_{th}^{2}\right)}^{1/2}$$

where n_ae_ is the aerodynamic deposition efficiency and n_th_ is the thermodynamic deposition efficiency.

The hygroscopic growth factor (G_f_) was calculated by [30,31,32]:

(4) $$G_{f}(RH)=\sqrt[{3}]{1+\frac{\kappa \times a_{w}}{1-a_{w}}}$$

where κ is the hygroscopicity parameter κ (0.3) and a_w_ is the water activity.

The water activity was calculated by [33,34,35]:

(5) $$a_{w}=\frac{RH}{100\times C_{k}}$$

where RH is the relative humidity and C_k_ is the Kelvin curvature correction factor.

The growth factor and the diameter of particles at a relative humidity of 99.5% were calculated using Equations (6) and (7), respectively [35,36]:

(6) $$G_{f}\left(99.5\%\right)=\sqrt[{3}]{1+\left(G_{f}^{3}\left(a\%\right)-1\right)\times \frac{99.5}{a}\frac{100\times C_{k}\left(a\%\right)-a}{100\times C_{k}\left(99.5\%\right)-99.5}}$$

(7) $$d_{99.5}=G_{f}(99.5\%)\times \frac{d_{a}}{G_{f}(a\%)}$$

where d_a_ is the diameter of particles at a% RH (before inhalation).

Finally, the particles deposited in the human respiratory tract were considered to be cleared due to the particle transport (nose blowing, mucociliary action) and absorption into the blood. The dose in the human respiratory tract after the clearance processes refer to retained dose. The retained dose of particles in the human respiratory tract and the dose to the esophagus, lymph nodes, and blood were also estimated based on the ICRP [29].

### 2.5. Health Risk Assessment Methodology

The hazard quotient (HQ) evaluates the non-carcinogenic risk caused by exposure to PM. An HQ value less than or equal to 1 indicates that the non-carcinogenic effects of PM are not of concern, while, an HQ value greater than 1 suggests that the non-carcinogenic effects of PM cannot be ignored. HQ was estimated by [37,38,39]:

(8) $$HQ=\frac{ADD}{RFD}=\frac{\left(C_{total}\times IR\times EF\times ED)/(BW\times AT\right)}{(RFC\times IR)/BW}=\frac{C_{total}\times EF\times ED}{RFC\times AT}$$

where ADD is the Average Daily Dose or intake (μg/kg/day), RFD is the reference dose (μg/kg/day), C_total_ is the pollutant concentration (μg/m^3^), IR is the inhalation rate (m^3^/d), EF is the exposure frequency (days/year), ED is the exposure duration (years), BW is the body weight (kg) of the exposed subject, AT is the averaging time (days) and RFC corresponds to a reference concentration (μg/m^3^).

The exposure duration (ED) was considered equal to 6 years (children value) based on US EPA classifications [38], while, the averaging time (AT) was considered equal to 2190 days (ED × 365 days/year) [38]. Furthermore, the exposure frequency (EF) was set equal to 180 days/year (number of school days per year in primary schools in Portugal) according to the OECD [40]. The RFD or RFC upper limit values of PM are not available and therefore the air quality guidelines levels (45 μg/m^3^ and 15 μg/m^3^ for PM_10_ and PM_2.5_, respectively) of WHO [22] were used as RFC.

## 3. Results and Discussion

### 3.1. PM Concentrations

Daily PM_10_ and PM_2.5_ concentrations in the indoor school environment (classroom) ranged from 22 to 161 μg/m^3^ and from 10 to 112 μg/m^3^, respectively. The corresponding concentrations in the indoor house environment ranged from 17 to 27 μg/m^3^ and from 12 to 22 μg/m^3^, respectively. Airborne particle concentrations at the monitoring station were lower than those observed at the indoor microenvironments (schools and houses). Specifically, the indoor PM concentration in the house microenvironment was 55.3% (PM_2.5_) and 8.6% (PM_10_) higher compared to those outdoors (Table 2). The monitoring station is located away from the city center and close to an area where the traffic is limited and therefore the vehicular traffic influence was limited. Moreover, PM_10_ and PM_2.5_ at School SA were at similar levels (the difference between PM concentrations of School SA and the station was less than 25% for 3/5 of days for both PM_10_ and PM_2.5_) to those measured at the monitoring station, while, for the remaining schools (which are located further away from the station) both PM_10_ and PM_2.5_ concentrations were higher (5/5 of days for school SB-SD).

The indoor PM concentrations (Table 2) in School SA were 17.7% (PM_2.5_) and 1.6% (PM_10_) higher than the ambient levels, while, in School SD it was 434.3% (PM_2.5_) and 502.2% (PM_10_) higher than the values measured at the monitoring station. It should be noted that chalk is used at School SD, whereas, at School SA whiteboard is used [25]. A comparison between the indoor and outdoor PM_2.5_ and PM_10_ concentrations at schools showed that indoor concentrations exceeded the outdoor ones (Table 2) indicating the importance of the indoor sources to the indoor particle concentration. Additionally, the highest concentrations among the measured schools were obtained in schools SD and SC. A comparison of indoor microenvironments showed that the highest indoor PM levels for both PM_10_ and PM_2.5_ were observed in schools and the lowest values were measured in homes.

All schools used chalk in traditional blackboards, except school SA which was equipped with whiteboards [25]. In addition, cleaning activities took place daily before the first course (09:00) [13]. The resuspension of particles due to the presence/movement of children and teachers is also an important indoor PM source [13]. Regarding ventilation, the schools have natural ventilation, and hence air renewal occurs by opening doors and single-glazing windows [25].

### 3.2. Ambient vs. Personal Regional Deposited Dose

**Figure 2:**
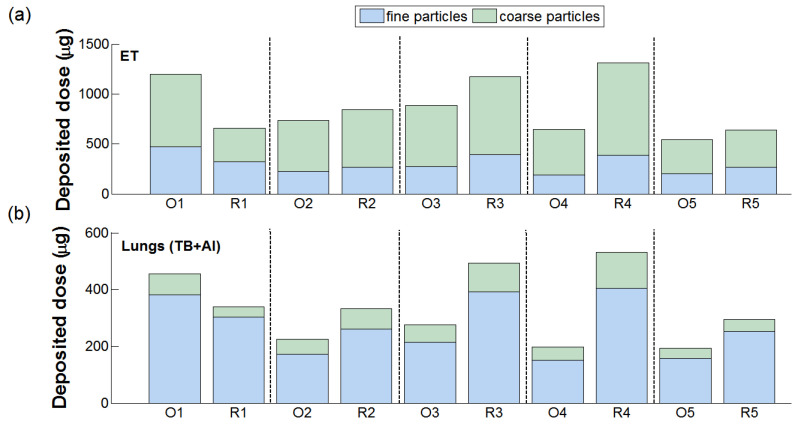
Weekly (5-days) deposited dose of fine (PM_2.5_) and coarse (PM_2.5–10_) particles at the (**a**) ET and (**b**) lungs (TB + AI) regions of 10-year-old children (O1–O5: Outdoor exposure scenario, R1–R5: Realistic exposure scenario).

The weekly ambient deposited dose of PM_10_ in the extrathoracic (ET) region assuming the outdoor exposure scenario (ambient dose) ranged from 545 μg to 1200 μg with an average value of 803 μg, while, the corresponding weekly personal deposited dose using the realistic exposure scenario (personal dose) ranged from 639 μg to 1311 μg with an average value of 925 μg (Figure 2a). In addition, a higher weekly deposited dose of PM_10_ in the ET region was obtained with the realistic scenario (R2–R5) in all cases except the first one (O1/R1). The latter is linked with the high ambient PM_10_ concentration (37.4 μg/m^3^) measured at the fixed monitoring station (O1). During the first case, although the ambient PM_10_ concentration was 39.4% higher than the personal PM_10_ concentration, the ambient deposited dose of PM_10_ in the ET was 82.1% higher than the personal deposited dose as a direct consequence of the higher contribution of PM_2.5–10_ to PM_10_ concentration. Specifically, the contribution of ambient PM_2.5–10_ to PM_10_ concentration during the first case (O1) was equal to 35.3% while the corresponding contribution of personal PM_2.5–10_ to PM_10_ concentration was equal to 23.0% (Table S2 in supplementary material).

Regarding the deposited dose in the lungs, the average weekly ambient deposited dose of PM_10_ was equal to 269 μg while the corresponding average weekly personal deposited dose was equal to 398 μg (Figure 2b). It was observed that 77–84% and 76–89% of the deposited dose in the lungs corresponds to fine particles in the case of ambient and personal doses, respectively. The dominance of fine particles in the lungs was also observed in other studies [41,42]. Aleksandropoulou et al. [41] found that 59% of the deposited dose in the lung region corresponds to fine particles for both ambient and personal doses, whereas, Sánchez-Soberón et al. [42] found that the deposited dose of coarse particles in the lungs was negligible. Faria et al. [14] that used direct methods (children carried a trolley with a portable monitoring device) for monitoring personal PM_2.5_ concentration found that the daily personal deposited dose of PM_2.5_ in the total respiratory tract varied between 97 to 177 μg whereas, in the current study, it varied between 85 to 258 μg. The daily personal deposited doses of PM_2.5_ reported by Faria et al. [14] were close to the results in the current study although they used direct methods and hence taking take into consideration all activities/microenvironments (e.g., sports in the gym and swimming pool) where children spent their time during the day.

### 3.3. Ambient vs. Personal Retained Dose and Clearance

**Figure 3:**
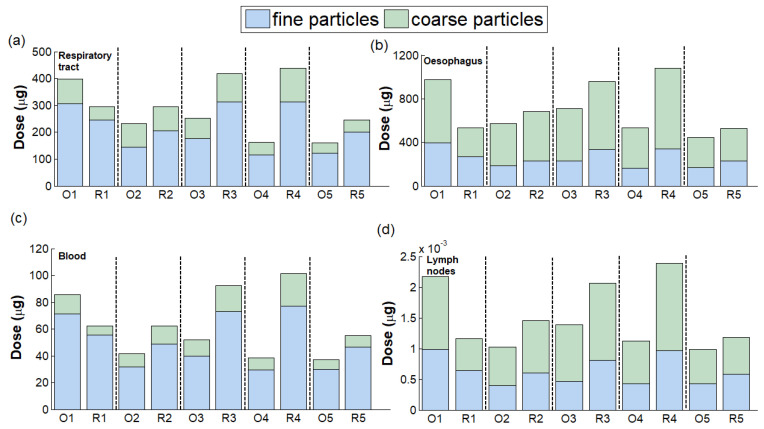
Weekly (5 days) dose of fine (PM_2.5_) and coarse (PM_2.5–10_) particles in the (**a**) respiratory tract (retained dose), (**b**) GI-tract (Oesophagus), (**c**) blood, and (**d**) Lymph nodes. O1–O5: outdoor exposure scenario, R1–R5: realistic exposure scenario.

The retained dose followed a similar variation with the deposited dose, therefore, the ambient retained dose was lower than the personally retained dose for all cases except the first one (O1/R1) (Figure 3a). Particularly, the ambient retained dose was 35.1% (PM_10_) and 24.4% (PM_2.5_) higher than the personally retained dose for the first case (O1/R1). In the other cases, the personally retained dose was 79.0% (PM_10_) and 88.9% (PM_2.5_) higher than the ambient retained dose. These results suggest that ambient data cannot be used for the calculation of personally retained dose for both PM_10_ and PM_2.5_ due to the underestimation of the dose in most cases. Similarly, the simulations for the dose in the GI tract, blood, and Lymph nodes match those of the retained dose (Figure 3b–d).

The majority of particles in the respiratory tract (Figure 3a) and blood (Figure 3c) were fine particles while in the Oesophagus (Figure 3b) and Lymph nodes (Figure 3d) were coarse particles. In more detail, 62–77% of the dose in the respiratory tract and 76–83% of the dose in blood corresponds to ambient fine particles, while, 59–69% of the dose to the esophagus and 54–67% of the dose to Lymph nodes corresponds to ambient coarse particles. Regarding the personal dose, 70–84% of the dose in the respiratory tract and 76–89% of the dose in blood corresponds to fine particles, while, 50–69% of the dose to the esophagus and 44–61% of the dose to Lymph nodes corresponds to coarse particles. These findings are associated with the deposition sites of fine and coarse particles. Coarse particles are deposited mainly in the ET region and are transferred more quickly to the esophagus, and hence they cause a greater effect on the digestive systems of children. On the other hand, fine particles can penetrate the lungs and remain in the respiratory tract or be absorbed into the blood which in turn causes a greater effect on potential lung diseases for children [42]. Particle size affects the deposition sites and therefore causes different health implications due to different clearance/translocate routes. The toxicity of particles depends on how quickly deposited particles can be removed or translocated which in turn depends on the deposition site [43,44]. Estimation of the dose taking into consideration the clearance processes is an essential step for evaluating health risks due to different behavior and effects of coarse and fine particles.

### 3.4. Linear Regression Analysis

**Figure 4:**
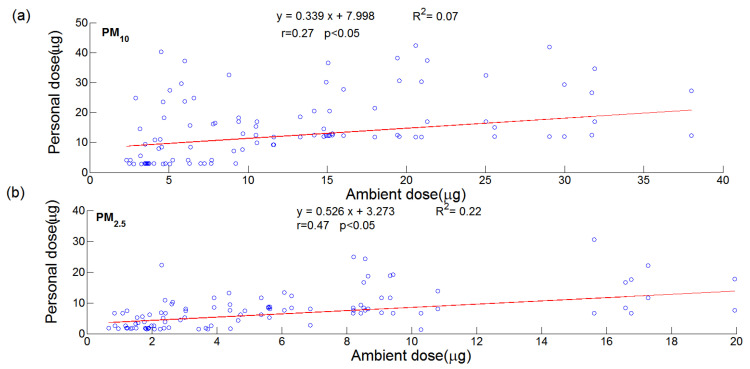
Linear regression between hourly ambient (outdoor exposure scenario) and personal (realistic exposure scenario) dose (μg) for (**a**) PM_10_ and (**b**) PM_2.5_.

Regression analysis between the hourly received doses (ambient vs. personal) showed statistically significant poor linearity with R^2^ equal to 0.07 and 0.22 for PM_10_ and PM_2.5_, respectively (Figure 4). However, linear regression results showed moderate strength under certain cases (see Figure S2 in supplementary material). Overall, increased R^2^ corresponds to the received dose from PM_2.5_ most likely associated with outdoor infiltrated particles indoors (Figure 4b and Figure S2b). In addition, the average relative deviation (ARD) was calculated. ARD between the ambient and the personal dose was equal to 0.60 and 0.51 for PM_10_ and PM_2.5_, respectively.

Faria et al. [14], that evaluated PM_2.5_ concentrations, found poor linearity (R^2^ = 0.31) between ambient and personal concentrations for schoolchildren in the metropolitan area of Lisbon. On the other hand, Chen et al. [45] found strong personal-ambient PM_2.5_ associations for students in Hong Kong with a Pearson correlation (r) equal to 0.73. Herein, the Pearson correlation between the ambient and the personal dose was equal to 0.27 and 0.47 for PM_10_ and PM_2.5_, suggesting low to moderate correlations respectively. These findings imply that particle mass concentrations and especially PM_10_ measured from fixed monitoring stations cannot be considered representative metric indicators for realistic exposure encountered by school children.

**Figure 5:**
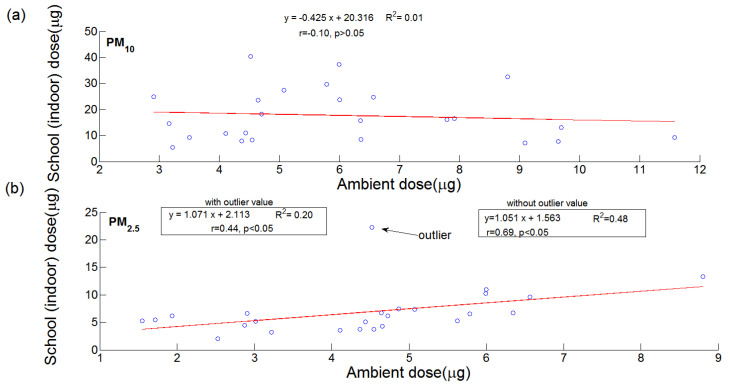
Linear regression between hourly ambient and school indoor dose (μg) for (**a**) PM_10_ and (**b**) PM_2.5_.

In addition, investigation of the linearity of the hourly received doses only between the ambient and the indoor school microenvironments showed again no linearity (R^2^ = 0.01; Figure 5a) for PM_10,_ but a moderate linear relationship was obtained for PM_2.5_ (R^2^ = 0.48; Figure 5b). The latter was associated with a strong positive correlation (r = 0.69), which indicates that the personal PM_2.5_ dose shares a common influence of the outdoor environment. In other words, the present results demonstrate that outdoor PM_10_ data are not sufficient representatives of the received indoor PM_10_ dose, whereas, outdoor PM_2.5_ data can be used as a predictor for the indoor PM_2.5_ dose in a school microenvironment. The ARD between ambient and school indoor doses was equal to 0.59 and 0.33 for PM_10_ and PM_2.5_, respectively. It was observed that the ARD for PM_2.5_ was lower in comparison to PM_10_, which indicates that ambient PM_2.5_ dose can be a better surrogate to represent the school indoor PM_2.5_ dose in comparison to PM_10_ dose data. In previous studies [7,46], authors found that the high PM_2.5_ and PN levels in schools were associated with outdoor sources, which indicates a high infiltration rate of particles and the absence of indoor sources. On the other hand, other studies [11,47] pointed out the importance of indoor sources to the high PM_10_ levels in school environments with dust resuspension representing a significant source of coarse particles. Both indoor and outdoor sources can influence PM_2.5_ concentrations in schools [48]. These observations underlie that taking outdoor data to estimate the personal exposure and dose should be conducted with care and a preliminary characterization of the under study environment is necessary in order to evaluate sources and inputs.

Almeida et al. [11] proposed the following control/mitigative/preventive methods for the management of assessed risks: good ventilation in order to remove particles, cleaning the blackboard with a damp cloth to avoid high concentrations of chalk particles suspended in the air, changing the type of board (from blackboard to whiteboard), prohibit smoking inside and near the buildings, selecting low emission products and vacuum cleaners/mops instead of brooms for cleaning activities, cleaning activities take place in the afternoon after the school hours. In addition, ventilation strategies such as closing the windows during rush hours or even changing the type of ventilation (from natural to mechanical ventilation) should be conducted to reduce the PM levels.

### 3.5. Impact of Hygroscopicity

**Figure 6:**
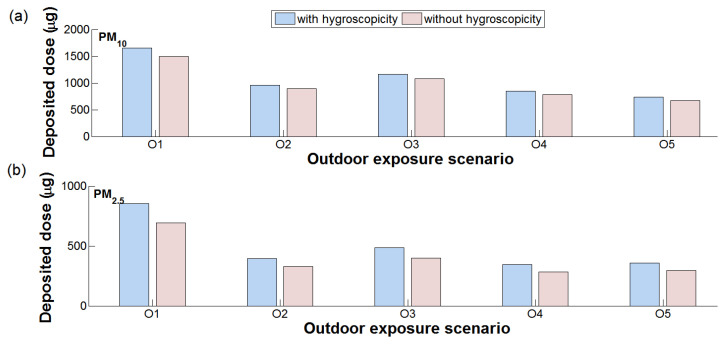
Weekly (5 days) deposited dose of (**a**) PM_10_ and (**b**) PM_2.5_ in the respiratory tract of children at the fixing monitoring station with and without the hygroscopic effect.

The incorporation of the hygroscopic growth of particles resulted in a higher deposited dose (Figure 6). Specifically, the deposited dose was increased by 8.8% and 21.7%, for PM_10_ and PM_2.5_, respectively. A similar observation was reported in Varghese and Gangamma [49] where the authors reported an increase of the deposited dose by 11% (PM_10_). Several studies [24,50,51] that examined the impact of hygroscopicity found that it tends to increase the total deposition fraction for particles greater than 0.2–0.3 μm therefore deposition occurs primarily via impaction and sedimentation mechanisms.

### 3.6. Hazard Quotients

The hazard quotients of both PM_10_ and PM_2.5_ were below the safe level (HQ < 1) for both scenarios (Table 3) which indicates that there is no increased risk for non-cancer health effects. However, the higher HQ obtained for PM_2.5_ -compared to PM_10_- implies a higher likelihood for non-carcinogenic hazards. It should be noted that results concern only the inhalation exposure route and school days thus they are indicative of the assumptions taken under consideration. Evidently, the cumulative hazard quotient incorporating all exposure pathways and periods is expected to give higher estimates, thus values above the safe level can be obtained. Madureira et al. [18] also estimated the HQ (PM_10_) for school children in primary schools and found values higher than 1. However, the authors have incorporated a considerably lower RFC (5 μg/m^3^), which influenced significantly the results. 

## 4. Conclusions

This study evaluated the use of ambient data for the calculation of the personal dose. It was found that the ambient dose cannot be used as a predictor for the personal dose for both PM_10_ (R^2^ = 0.07) and PM_2.5_ (R^2^ = 0.22). On the other hand, the ambient dose can be used with caution as a predictor for the (school) indoor dose of PM_2.5_ (R^2^ = 0.48 and r = 0.69). The values of R^2^ and r were higher for PM_2.5_ than PM_10_ and hence the correlation between personal or school indoor dose and ambient dose was higher for PM_2.5_ than PM_10_. The novelty/advantage of the current study is the use of linear regression analysis on PM doses whereas previous studies in the scientific literature focused on PM concentrations. Understanding the representativeness of ambient PM data for the determination of personal dose is important for controlling air pollution in the school environments and improvement of children’s health. The limitation of the current study is that the set of data was limited (5 days for each school) for accurate estimations of the representativeness of ambient data and hence the findings must be used with caution for other cases.

In addition, the average weekly ambient dose was lower than the average weekly personal dose for both PM_10_ and PM_2.5_. The average weekly ambient dose in the respiratory tract was equal to 1072 μg for PM_10_ and 487 μg for PM_2.5_ while the corresponding personal dose was equal to 1323 μg for PM_10_ and 649 μg for PM_2.5_, which implies that the ambient dose underestimates the personal dose. The representativeness of ambient data for the estimation of the personal dose depends on the school location, PM physicochemical characteristics, and source strengths. However, the findings of the study must be used with caution since they apply to a specific city and school location. The hazard quotient that represents the non-carcinogenic effects due to the inhalation was lower than the safe level (<1) and hence no significant risk is expected for all the scenarios investigated. Although hazard quotients were lower than the safe level, the cumulative hazard quotient taking into consideration all exposure pathways and periods is expected to give higher estimates. Therefore, mitigation strategies should be conducted to reduce the PM levels in school environments.

## Figures and Tables

**Table 1:** Sampling period for each school and respective exposure scenario (realistic/outdoor) for each case.

Field Measurements		Scenarios	
Sampling Period	Location	Outdoor	Realistic
16–17/11/17 & 20–22/11/17	School SA	station (O1)	daily activity profile (R1)
23–24/11/17 & 27–29/11/17	School SB	station (O2)	daily activity profile (R2)
7/12/17 & 11–14/12/17	School SC	station (O3)	daily activity profile (R3)
15–19/01/18	School SD	station (O4)	daily activity profile (R4)
21–25/05/18	School SE	station (O5)	daily activity profile (R5)

**Table 2:** Average (±standard deviation) of PM_10_ and PM_2.5_ concentrations (μg/m^3^) measured in schools, houses, and Olivais air quality monitoring stations. The measuring period corresponds to 8 h at schools and 15 h at the houses.

Location	PM_2.5_		PM_10_	
	Indoor	Outdoor	Indoor	Outdoor
School SA	28.6 ± 6.0	26.0 ± 4.4	38.0 ± 8.5	37.1 ± 4.2
School SB	23.6 ± 8.1	9.7 ± 7.1	51.6 ± 8.4	20.7 ± 9.5
School SC	48.3 ± 13.4	27.9 ± 11.2	89.7 ± 21.6	45.0 ± 9.0
School SD	52.9 ± 34.1	19.7 ± 12.1	109.0 ± 34.3	28.3 ± 11.4
School SE	19.5 ± 3.6	18.0 ± 10.5	32.7 ± 7.5	25.2 ± 13.3
House *	16.0 ± 3.6	-	20.2 ± 3.8	-
Station (O1)	-	24.3 ± 4.7	-	37.4 ± 7.3
Station (O2)	-	11.5 ± 3.1	-	20.8 ± 7.9
Station (O3)	-	14.0 ± 6.1	-	25.1 ± 6.8
Station (O4)	-	9.9 ± 4.3	-	18.1 ± 4.5
Station (O5)	-	10.4 ± 2.4	-	16.6 ± 2.5
Station **	-	10.3 ± 1.2	-	18.6 ± 1.3

* average of 34 houses. ** average of September 2017–July 2018 (sampling period of houses).

**Table 3:** Hazard quotients of PM_10_ and PM_2.5_ during outdoor (O) and realistic (R) exposure scenarios.

	HQ		HQ
PM_10_			
O1	0.41	R1	0.29
O2	0.23	R2	0.34
O3	0.28	R3	0.49
O4	0.20	R4	0.55
O5	0.18	R5	0.27
PM_2.5_			
O1	0.80	R1	0.68
O2	0.38	R2	0.60
O3	0.46	R3	0.90
O4	0.33	R4	0.94
O5	0.34	R5	0.57

## Data Availability

The data presented in this study are available on request from the corresponding author.

## Supplementary Materials

The following supporting information can be downloaded at: https://www.mdpi.com/article/10.3390/ijerph20085564/s1, Table S1: Daily activity profile during weekdays; Table S2: Average (±st. deviation) contribution (%) of PM_2.5–10_ (μg/m^3^) to PM_10_ (μg/m^3^) concentration; Figure S1: Size distribution of the mass fraction of PM_2.5_ for three microenvironments (house-indoor, school-indoor, and school-outdoor). The outdoor values were used for the fixed monitoring station; Figure S2: Linear regression results of hourly ambient (outdoor exposure scenario) and personal (realistic exposure scenario) dose (μg) of (a) PM_10_ and (b) PM_2.5_ in the human respiratory tract of 10-year-old child.
