# Supplementary material for: The Representativeness of Outdoor Particulate Matter Concentrations for Estimating Personal Dose and Health Risk Assessment of School Children in Lisbon
Source: Int J Environ Res Public Health. 2023 Apr 18;20(8):5564. doi: 10.3390/ijerph20085564 (PMC10138915; doi:10.3390/ijerph20085564)
Supplement: Supplementary file 1 [file ijerph-20-05564-s001.zip › ijerph-2314794-supplementary.pdf]

Supplementary material

Table S1. Daily activity profile during weekdays.

Time	Activity	Microenvironment
00:00 - 08:00	Sleep	House (indoor)
08:00-09:00	Light exercise	House (indoor)
09:00-13:00	Sitting	School (indoor)
13:00-14:00	Light exercise	School (outdoor)
14:00- 18:00	Sitting	School (indoor)
18:00- 22:00	Light exercise	House (indoor)
22:00-00:00	Sleep	House (indoor)

Table S2. Average (±st. deviation) contribution (%) of $PM_{2.5-10}$ ($\mu g/m^3$) to PM_{10} ($\mu g/m^3$) concentration.

Scenarios/subcases	Outdoor exposure scenario (O)	Realistic exposure scenario (R)
1	35.3±1.4	23.0±2.9
2	42.2±9.5	39.5±11.5
3	46.1±10.0	38.4±6.5
4	45.9±17.1	44.4±9.7
5	37.9±6.6	28.9±5.9

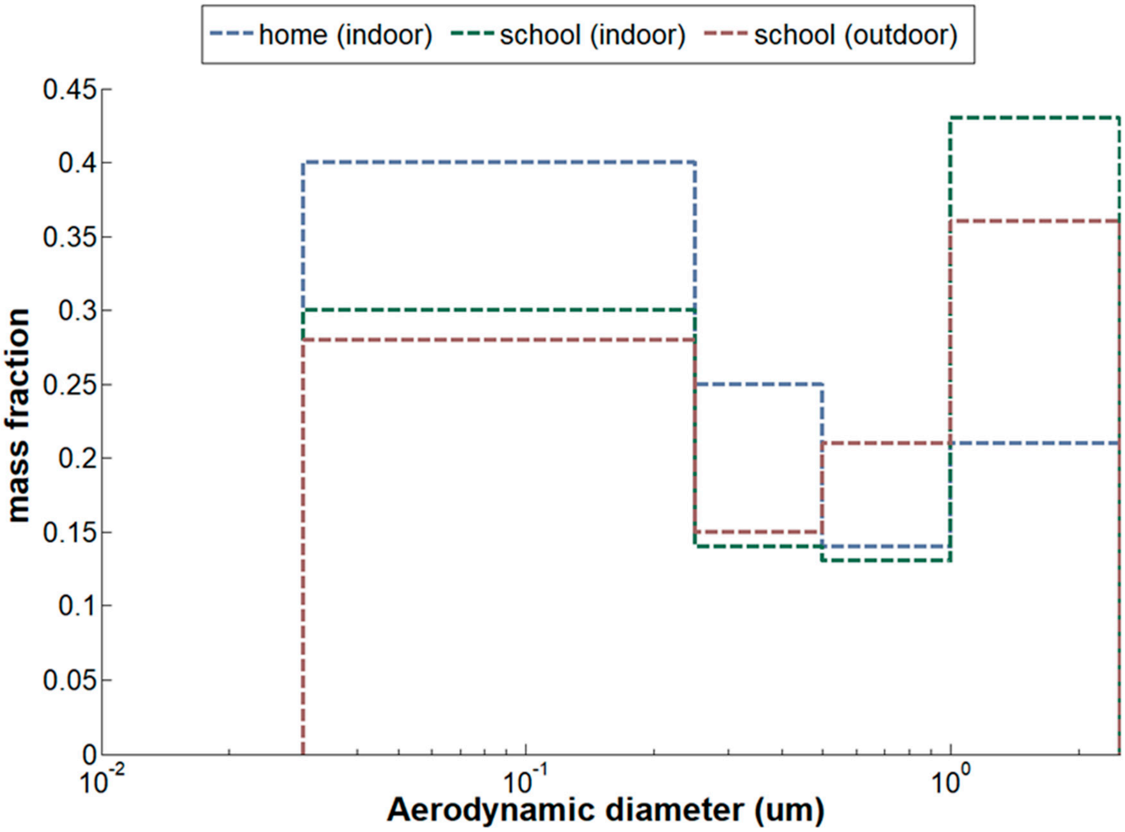

Figure S1. Size distribution of the mass fraction of $PM_{2.5}$ for three microenvironments (house-indoor, school -indoor and school -outdoor). The outdoor values were used for the fixed monitoring station.

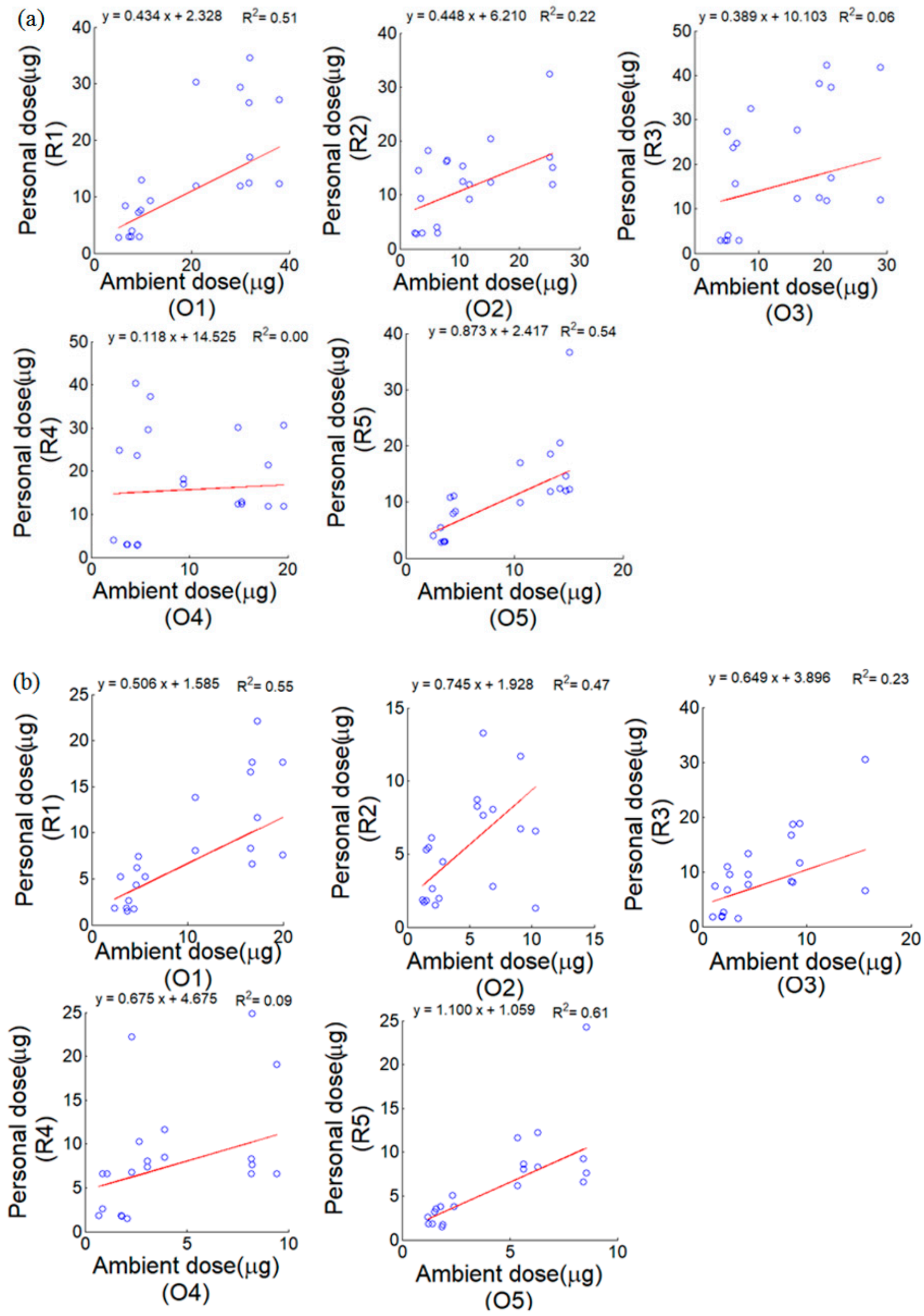

Figure S2. Linear regression results of hourly ambient (outdoor exposure scenario) and personal (realistic exposure scenario) dose (μg) of (a) PM_{10} and (b) $PM_{2.5}$ in the human respiratory tract of 10 years old child.
